# Mechanism and control technology of roadway floor heave deformation and failure under the influence of repeated mining in multiple coal seams

**DOI:** 10.1038/s41598-025-16336-3

**Published:** 2025-08-26

**Authors:** Fu Yuping, Ren Yiming, He Yongliang, Li Chuantian

**Affiliations:** 1 School of Safety and Emergency Management Engineering, Taiyuan University of Science and Technology, 030024 Taiyuan, Shanxi People’s Republic of China; 2 Intelligent Monitoring and Control of Coal Mine Dust Key Laboratory of Shanxi Province, Taiyuan University of Science and Technology, 030024 Taiyuan, Shanxi People’s Republic of China

**Keywords:** Multiple seam, Floor heave, Roadway damage, Control technology, Destructive zone, Engineering, Civil engineering

## Abstract

Deformations caused by of roadway floor heave due to the repeated mining of multiple coal seams is a prominent problem in deep coal mining. This work examines roadway floor heave under the influence of repeated mining in multiple coal seams as the research object. Through theoretical analysis, numerical simulation, and field experiments, the failure mechanism of floor heave deformation is revealed, and effective control techniques are proposed. A deformation model for multiple-seam repeated mining was established based on the theory of elastic mechanics, and a control mechanism was analysed. An optimised treatment method for roadway floor heave was proposed. Displacement, stress, and failure zone analyses of the original support and optimised surrounding rock were conducted via FLAC^3D^ software, and the optimal treatment method for roadway floor heave was obtained. The applicability of this support method was verified through onsite application. The results indicate that stress superposition is the cause attributed to the repeated mining of multiple coal seams, and stress reduction and effective roof support are the keys to preventing and controlling roadway floor heave. Compared with the original support method, the amount of floor heave was reduced by 60%, and the shrinkage of the two sides was also significantly reduced. The optimised roadway support method provides better control over floor heave.

## Introduction

With the continuous increase in global energy demand, coal, as an important energy resource, still holds a significant position in the energy structure of many countries^1,2^. To improve the mining efficiency and economic benefits of coal mines, an increasing number of mines are adopting the method of layered extraction of multiple coal seams^3,4^. However, the problem of floor heave deformation caused by the repeated mining of multiple coal seams has become a significant factor affecting the safe production and economic efficiency of coal mines. The stability of underground roadways is a critical challenge in coal mining, particularly under the influence of repeated mining of multiple coal seams^5,6^. As shallow coal resources are gradually depleted, deep mining and collaborative development of multiple coal seams have become inevitable trends in the global coal industry. The repeated mining of multiple coal seams can lead to complex stress redistributions and cumulative damages in the surrounding rock, resulting in severe floor heave in roadways^7^. This phenomenon not only affects the basic functions of ventilation, transportation, and equipment installation but also induces significant safety hazards such as roof collapses and support failures. Although substantial research has been conducted on roadway stability, the deformation mechanisms and control strategies for floor heave under the conditions of repeated mining of multiple coal seams have to be sufficiently addressed and require further exploration.

The formation of floor heave is jointly controlled by multiple factors, including high ground stress, weak floor strata, groundwater seepage, and disturbance from mining-induced stress^8^. In single coal seams, scholars have proposed mechanisms such as stress-driven plastic flow, soft rock rheological expansion, and the buckling instability of layered floors and have developed control technologies, including reinforced concrete underpinning, anchor grouting reinforcement, and pressure relief grooves. Wen Zhijie^9^ established a soft rock roadway floor heave model, analysed the floor heave control mechanism, and proposed optimised support methods. The modification of the rock surrounding the roadway and effective support are the keys to soft rock roadway floor heave control. Sungsoon Mo^10^ introduced a classification method for floor heave failure mechanisms, which classifies roadway floor heave on the basis of the floor heave index. The determination of floor heave conditions depends on the coal mine floor level and horizontal stress level. Ivan Sakhno^11^ studied the mechanism of floor heave and grouting control technology in soft rock roadways with respect to the water content. As the water content increases, the compressive strength and friction angle of mudstone decrease linearly, whereas the deformation modulus and cohesion exhibit an approximately negative exponential decreasing trend. Wang Tie^12^ simulated the deformation and failure process of soft rock roadways in high-humidity environments. During the initial excavation stage, only the surface humidity of the surrounding rock changed significantly, and the floor heave caused by humidity was small but varied greatly. The environmental humidity significantly affects the structural performance of soft rock roadways. Qingwen Zhu^13^ studied the rheological process of roadways and reported that the large deformation zone of the surrounding rock continuously shifts from the surface to greater depths. After the deformation of the anchorage zone, the bearing capacity of the anchor rod rapidly decreases. The mechanical properties of the floor heave indicate that the lower surrounding rock always repeats the failure process of the upper rock layer. Youlin Xu^14^ analysed the changes in stress, displacement, and plastic zones around roadways during the upper coal seam mining process, as well as the causes of roadway deformation. With increasing mining in the upper working face, the vertical stress, maximum shear stress, and plastic zone of the rock surrounding the roadway increase. The deformation and failure of roadways mainly occur in the floor heave, sides, and corners of roadways. Penghai Deng^15^ studied the failure process of floor rock masses via the finite discrete element method (FDEM) and proposed a mechanical mechanism for floor uplift that considers the effects of geostress, lateral coefficient, and tensile strength. Guojun Wu^16^ proposed an improved roadway support scheme that uses long cables to suppress severe deformation of the roadway and short cables to seal U-shaped steel groups to improve the stress state of the roadway. Compared with the original plan, this approach can effectively suppress floor heave and control significant asymmetric deformation. Qingwen Zhu^17^ studied the creep deformation and failure characteristics of a soft rock roadway under joint support conditions. Under high stress, the soft rock roadway tends to experience continuous failure and nonconvergence, with the cross-sectional area decreasing to 52.3% of that of the original roadway.

Scholars have extensively discussed the causes of floor heave deformation, primarily focusing on stress concentration, and hydrogeological conditions^18–20^. Research has focused mostly on single coal seams or local areas, and systematic studies on the deformation of floor bulges during multiple seam mining processes are lacking. During the repeated mining process of multiple coal seams, the overlying rock undergoes progressive damage under cyclic loading and unloading, leading to intensified stress concentration effects and accelerated degradation of the mechanical properties of the floor rock mass. Research often overlooks the dynamic evolution laws of stress fields and cumulative damage effects under superimposed mining disturbances, resulting in insufficient accuracy in predicting deformation and a lack of theoretical support for optimising support schemes.

This study uses theoretical analysis, numerical simulation, and field experiments to systematically reveal the deformation and failure mechanism of roadway floor heave induced by the repeated mining of multiple coal seams. By analysing the dynamic stress evolution law and plastic zone expansion through numerical simulation, a collaborative control technology of"floor pressure relief + passive support"is proposed to alleviate high floor stress while improving roadway stability. Good results were achieved using this control technology in field applications.

## Geological and destructive conditions

### Geological overview

The Shaqu No.1 Mine, which is under the jurisdiction of the Huajin Coking Coal Co., Ltd., is located in the middle section of the Liliu Mining Area in Hedong Coalfield, Shanxi Province, on the west wing of the Lvliang Mountain Anticline. Its geographical location is west of Liulin County, Shanxi Province, and the industrial site is approximately 5 km away from the county. At present, four mining areas have been formed underground in the northern wing via the original main roadway. The #4 and #5 coal seams in the first mining area have been largely mined, and the #2 coal seam in the second mining area is partially mined as a protective layer. The #4 coal seam in the second mining area has been partially mined, whereas the #5 coal seam in the third mining area has not been mined. The 5301 working face of coal seam #5 is being mined, with a mining height of approximately 2.8 m and a working face length of 200 m. The coal is being mined using the inclined longwall one-time full-height coal mining method, and the full collapse method is being used to manage the roof. No working face exists in the fourth mining area.

**Fig. 1:**
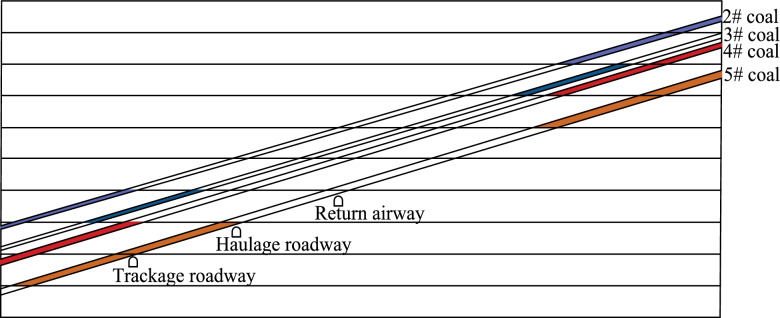
Stratigraphic relationships between roadways and coal seams.

The horizontal transportation roadway, track roadway, and return air roadway of the mine + 400 m are located in the bottom rock layer of the #5 coal seam. After being affected by the dynamic pressure of the #2 coal seam mining face and #4 coal seam mining face, they are also affected by the repeated mining of the #5 coal seam mining face. Onsite research has revealed that the main causes of rock damage in transportation, track, and return air roadways are rapid deformation, severe floor heave, and compression of two sides. Owing to the concentrated stress of residual coal pillars in the overlying working face and the impact of mining in the current coal seam working face, stress is superimposed and acts on the rock surrounding the main roadway, causing the section of the main roadway to shrink and causing floor heave. The maximum amount of floor heave in some areas of the main roadway is up to 1000 mm, and the maximum range of floor heave is 300–400 mm, causing severe deformation and damage to the roadway. The main roadway needs to be repeatedly repaired, which seriously affects the safety of coal transportation, material transportation, and pedestrians in the main roadway. Owing to the shrinkage of the section of the main roadway, ventilation is unfavourable, which severely affects the safety and efficient production of the mine. The relationship between the roadway and the coal seam is illustrated in Fig. 1.

### Coal seam histogram

The mining depth of coal seams in the Shanxi Formation is relatively deep, and early coal mining has mainly occurred in shallow layers below the surface. The harm caused by geostress was not a significant problem in early mining. With increasing underground mining depth, the deformation caused by high ground stress, such as delamination and sliding, affects the rock surrounding the roadway, leading to increasingly prominent problems such as roof subsidence, floor heave, and anchor fractures. Roof caving accidents also occur frequently. Joint support control technology with anchors has a significant inhibitory effect on the discontinuous deformation of rock surrounding roadways but has little effect on controlling continuous large deformation of surrounding rock deep in roadways.

**Fig. 2:**
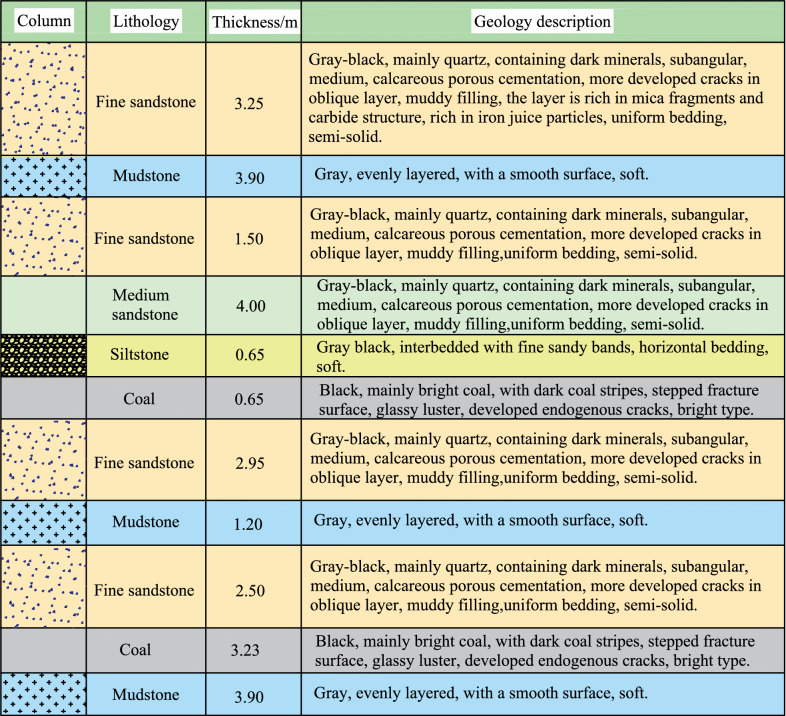
Coal seam histogram.

The coal-bearing strata in the field are the Shanxi Formation and Taiyuan Formation. A total of 15 coal seams exist, among which #1–#5 are produced in the Shanxi Formation. The coal seam structure is shown in Fig. 2. The exploitable coal seams in the Shanxi Formation are coal seams #2, #3, #4 and #5, with average interlayer spacings of 16.44, 4.2, and 4.45 m, respectively. The geological conditions of the main alley are simple, with a weak water content in the fault and no water conductivity. The main water source is fissure water, and water inflow mainly occurs in the form of dripping water. The direct roof rock layer in the main alley is approximately 2.2–11.4 m thick and is composed of thin layers of fine-grained sandstone and coal seams. The floor heave is composed of fine-grained sandstone, coal, and mudstone, with a thickness of approximately 5.5–9.16 m. The floor heave has poor integrity and low strength, and the rock properties of each layer are not significantly different. The bearing capacity of the floor heave is low, making it difficult to form a stable structure, and under high stress, the floor heave exhibits significant deformation characteristics.

### Roadway floor heave damage

Unlike single-seam coal mining^21^, mining of an upper coal seam will generate stress concentration and transmission at the coal seam floor and protective coal pillars in multiple-seam mining. The stress is transmitted downwards through the protective coal pillars and rock layers, and the superimposed stress formed by the mining of multiple coal seams is transmitted to the rock surrounding the roadway, causing a change in its stress state. The change in the stress state is an important influencing factor leading to roadway floor heave^22^.

The main coal seams that can be mined in the mine are coal seam #2, coal seam #3 + 4, and coal seam #5. The + 400 m horizontal track roadway studied in this article is located in the bottom rock layer of coal seam #5. After being affected by the dynamic pressure of the #2 and #4 coal seam working faces and then by the repeated mining of the #5 coal seam working face, the superimposed stress generated during the multiple-seam mining process is transmitted to the rock surrounding the main roadway, causing a change in the stress state of the rock surrounding the main roadway. Moreover, because the average distance between the #3 + 4 coal seam and the #5 coal seam is only 4.45 m, the roadway can be characterised as a close-range coal seam group. For close-range coal seam mining, the superimposed stress generated by mining severely affects the stress distribution of the rock mass on both sides of the roadway. The stress concentration coefficient of the two sides of the roadway tends to increase. Under the high peak stress concentration phenomenon formed by the two sides of the roadway, the roadway is prone to floor heave.

**Fig. 3:**
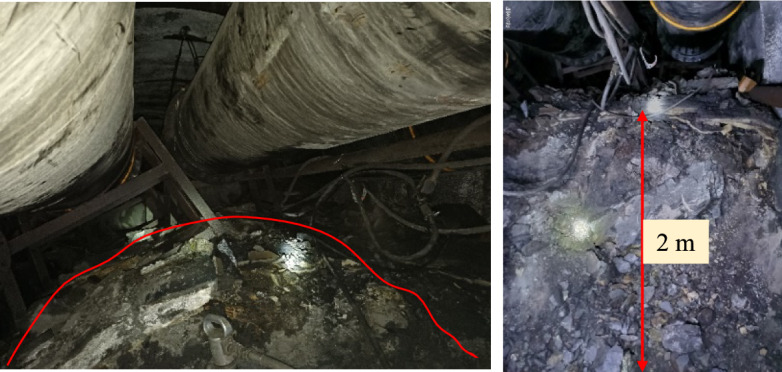
Roadway floor heave damage.

The phenomenon of floor heave causes a reduction in the cross-section of the roadway, hindering equipment transportation and personnel movement, affecting the normal mining of the working face, and increasing maintenance work and maintenance costs^23^. A floor heave is typically arc-shaped, with a higher middle and lower sides, which makes transit difficult^24^. The mine only keeps raising the bottom, which further deteriorates the lithology of the floor, resulting in stronger floor heave of the roadway when affected by the mining of this working face, making it difficult to meet the safety standards, as shown in Fig. 3. The maximum floor heave can reach 2 m. After excavation, the cracks in the surrounding rock mass are disturbed and further expanded, eventually forming a loose circle. The degree of development of the rock mass loosening zone is closely related to the physical and mechanical properties of the rock mass, the stress distribution state of the rock mass, and the strength of the rock support. As the working face continues to advance, the mining stress on the surrounding rock continues to increase, and the internal structure of the surrounding rock becomes more severely damaged. The loosening zone is also amplified, leading to instability and failure of the rock surrounding the roadway. At this point, grouting can consolidate the broken rock together into large blocks to improve the stability of the surrounding rock.

## Theoretical basis of floor heave in multiple-seam mining

**Fig. 4:**
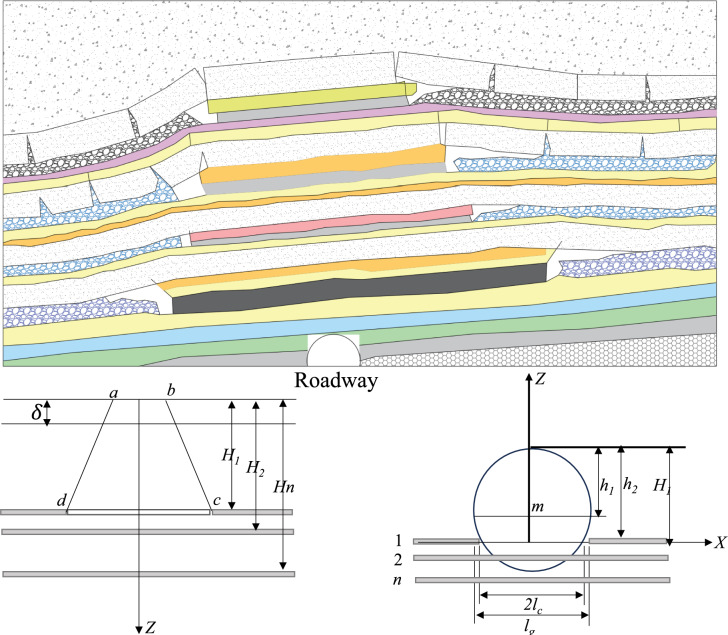
Simplified model.

The roadway floor is assumed to be a homogeneous isotropic elastic medium, ignoring the inhomogeneity of the middling coal body during coal mining in Fig. 4. The deformation of the roadway floor is affected mainly by mining stress, ignoring factors such as temperature and seepage^25^. The roadway is excavated along the horizontal plane, and the mining of each coal seam affects the bottom of the roadway during the mining process. The mining of each layer of coal causes a redistribution of stress in the overlying rock layers and affects the roadways below. The roadway has a planar geometric shape, and the deformation caused by floor heave is concentrated mainly at the bottom of the roadway. The principle of stress superposition in elastic mechanics is used to analyse the floor heave deformation of each coal seam after mining^26^.

The initial stress state $\sigma_{0}$ is assumed to be a uniform static stress field:

1 $$\sigma_{0} = \sigma_{0xx} = \sigma_{0yy}$$

(1) 1 st layer coal mining.

The stress change caused by the mining of the first layer of coal is $\Delta \sigma_{1}$ The stress field after the mining of the first layer is:

2 $$\sigma_{1} = \sigma_{0} + \Delta \sigma_{1}$$

The stress change $\Delta \sigma_{1}$ can be expressed as:

3 $$\Delta \sigma_{1} = \sigma_{xx1} + \sigma_{yy1}$$

Deformation of floor heave after the first layer of mining is:

4 $$\delta_{1} = \frac{{\sigma_{1} h^{3} }}{12EI}$$

where $\delta_{1}$ is the deformation displacement of the first layer of mining, m; $h$ is the height of the roadway, m; $E$ is the elastic modulus; and $I$ is the moment of inertia.

(2) 2nd layer coal mining.

The stress change caused by the mining of the first layer of coal is $\Delta \sigma_{2}$ The stress field after the mining of the second layer is:

5 $$\sigma_{2} = \sigma_{1} + \Delta \sigma_{2} = \sigma_{0} + \Delta \sigma_{1} + \Delta \sigma_{2}$$

The stress change $\Delta \sigma_{2}$ can be expressed as:

6 $$\Delta \sigma_{2} = \sigma_{xx2} + \sigma_{yy2}$$

Deformation of floor heave after the second layer of mining is:

7 $$\delta_{2} = \frac{{\sigma_{2} h^{3} }}{12EI} = \frac{{\left( {\sigma_{0} + \Delta \sigma_{1} + \Delta \sigma_{2} } \right)h^{3} }}{12EI}$$

(3) *n*th layer coal mining.

When mining the *n*th layer of coal, the stress change is $\Delta \sigma_{n}$, and the stress field at this time is:

8 $$\sigma_{n} = \sigma_{n - 1} + \Delta \sigma_{n} = \sigma_{0} + \sum\limits_{i = 1}^{n} {\Delta \sigma_{i} }$$

The stress change $\Delta \sigma_{n}$ can be expressed as:

9 $$\Delta \sigma_{n} = \sigma_{xxn} + \sigma_{yyn}$$

Deformation of floor heave after the *n*th layer of mining is:

10 $$\delta_{n} = \frac{{\sigma_{n} h^{3} }}{12EI} = \frac{{\left( {\sigma_{0} + \sum\nolimits_{i = 1}^{n} {\Delta \sigma_{i} } } \right)h^{3} }}{12EI}$$

Considering the cumulative effect of mining all the coal seams on the deformation of floor heave, the total deformation can be expressed as:

11 $$\delta_{total} = \sum\limits_{i = 1}^{n} {\delta_{i} } = \sum\nolimits_{i = 1}^{n} \delta = \sum\limits_{i = 1}^{n} {\frac{{\left( {\sigma_{0} + \sum\nolimits_{j = 1}^{i} {\Delta \sigma_{i} } } \right)h^{3} }}{12EI}}$$

The total deformation of floor heave in the roadway is caused not only by stress changes during single-seam coal mining but also by the comprehensive result of the superposition effect of multiple-seam coal mining. According to the principle of stress superposition in elasticity, the total deformation can be expressed as:

12 $$\delta_{total} = \sum\limits_{i = 1}^{n} {f\left( {\sigma_{0} + \sum\nolimits_{j = 1}^{i} {\Delta \sigma_{i} } } \right)}$$

where $f\left( {\sigma_{0} + \sum\nolimits_{j = 1}^{i} {\Delta \sigma_{i} } } \right)$ is the relationship function between stress and deformation, usually linear or nonlinear, depending on the geometric shape, material properties, and loading method of the tunnel. The specific stress changes are obtained through the elastic response of the rock layer.

For the case of multiple-seam coal mining, the total deformation $\delta_{total}$ at the bottom of the roadway can be expressed in the form of stress integration to consider the complex stress transmission and influence between coal seams. By using the integration formula, we obtain:

13 $$\delta_{total} = \int_{0}^{n} {\frac{{\left( {\sigma_{0} + \sum\limits_{i = 1}^{n} {\Delta \sigma_{i} } } \right)h^{3} }}{12EI}} dn$$

where the range of integration from 0 to *n* represents the stress superposition from the 1 st coal seam to the *n*th coal seam.

The mining of coal seams not only has static effects but also may be accompanied by dynamic effects, which are particularly evident in mining operations. Dynamic effects may lead to additional stress changes $\Delta \sigma_{dyn}$, which need to be considered:

14 $$\Delta \sigma_{dyn} = \sum\limits_{i = 1}^{n} {\Delta \sigma_{dyni} }$$

This will cause additional deformation $\delta_{dyn}$ at the bottom of the roadway, resulting in a total deformation of:

The stress changes after each layer of coal mining will be superimposed on the bottom of the roadway, leading to the accumulation of deformation. The relationship between deformation and stress can be calculated through elastic mechanics formulas, and the deformation of floor heave is the result of stress superposition. Factors such as dynamic effects, stress transfer, and pore water pressure may affect the deformation of floor heave and need to be considered in practical analyses. When the stress exceeds the critical value of failure, the bottom floor may fail, and the structural stability needs to be judged according to the failure criteria.

As the depth and number of layers of close-range multiple-seam mining increase, the stress concentration of the remaining coal pillar floor gradually increases, corresponding to the stress concentration around the roadway. As the vertical stress on the two sides of the roadway gradually increases, the roadway floor gradually bends and breaks, and the greater the vertical stress on the two sides of the roadway is, the greater the depth of damage to the roadway floor. Subsequently, under high stress concentrations around the roadway, the fractured rock gradually bulges in the direction of the roadway overhang. Therefore, in response to the complex mining environment of the Shaqu Coal Mine, analysing the impact of coal seam mining on the stress concentration of the rock surrounding the roadway is crucial for roadway maintenance. The establishment of this model has important practical significance for the analysis of the stability of the rock surrounding the roadway in the coal seam layer of the coal pillar floor under the conditions of close-range coal seam mining.

## Characteristics of overlying rock structure migration during the repeated mining of multiple coal seams

To facilitate the study of the deformation law of floor heave of the main roadway under multiple-seam mining, a model was established via FLAC^3D^ software to simulate the deformation and damage of the rock surrounding the main roadway under the sequential mining of coal seams #2, #3 + 4, and #5. At the same time, monitoring points were applied to both sides and the roof and floor of the roadway to obtain the stress and displacement deformation of the surrounding rock under multiple-seam mining. The FLAC^3D^ numerical calculation model was established on the basis of the engineering geological conditions of the mine and the characteristics of the roof and floor rock layers of the tunnel. The Mohr–Coulomb model was selected as the numerical model, considering boundary effects. The length is 240 m, the width is 240 m, and the height is 90 m. Cable commands were used for anchor rods and cables. The physical and mechanical parameters of the rock are shown in Table 1. Research has been conducted on different support and pressure relief methods, analysing the displacement changes in the top support, floor heave reinforcement support, and pressure relief groove. On the basis of boundary conditions, the normal displacement of the front, back, left, right, and model bottom was restricted.

**Table 1:** Physical and mechanical parameters of rocks.

Rock type	Density g/cm^3^	Bulk modulus/GPa	Shear modulus/GPa	Internal friction angle/(°)	Cohesion/MPa	Compressive strength/MPa
Mudstone	2.59	0.82	0.47	42	2.0	3.05
Sandy mudstone	1.82	1.60	0.49	42	1.30	0.18
#2 coal	1.30	1.00	0.50	36	1.80	0.80
Medium sandstone	1.69	3.68	1.05	37	4.78	1.50
Fine sandstone	2.60	6.00	4.00	15	2.00	1.50
#3 coal	1.36	1.01	0.58	40	1.82	0.77
Siltstone	1.76	7.00	6.00	30	3.230	0.38
#4 coal	1.36	1.01	0.58	40	1.82	0.77
#5 coal	1.36	1.01	0.58	40	1.82	0.77

Roadway roof support.

**Fig. 5:**
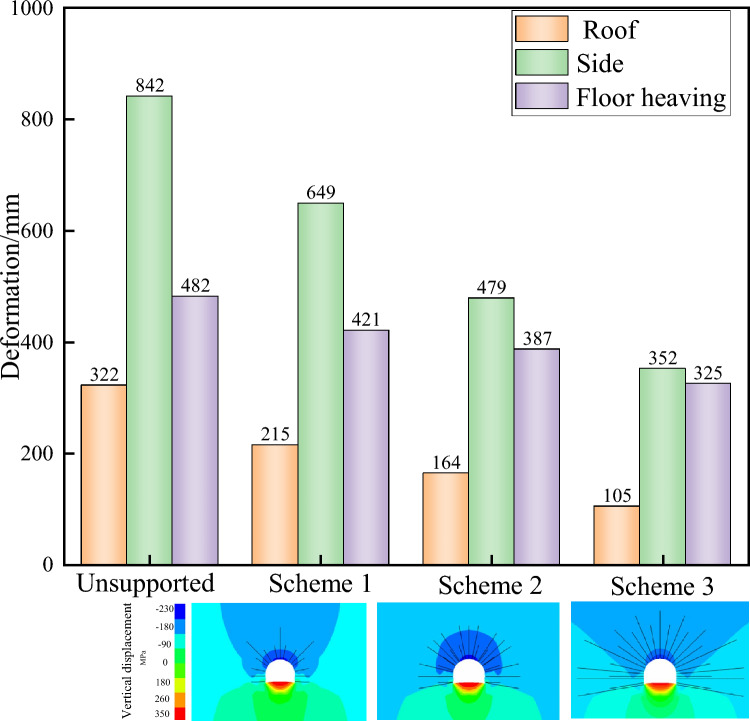
Displacement under different top supports.

A comparison of the support effects under different top support conditions, as shown in Fig. 5 and Table 2, reveals that with increasing strength of the roadway roof and two side supports, the displacement of the roadway significantly decreases. The corresponding bottom bulge amounts for schemes 1 to 3 are 421, 387, and 325 mm, which are 12.6%, 19.7%, and 32.6% lower than those without support (482 mm), respectively. However, owing to the unsupported state of floor heave, the displacement of the bottom angle increases. Therefore, the top support plays a role in controlling the deformation and failure of the floor heave to a certain extent but increases the degree of deformation and failure of the floor heave. Therefore, under the condition of top support, the bottom support should be strengthened to form a complete load-bearing body^27^.

**Table 2:** Different support schemes for roof support.

Scheme	Simulation diagram	Describe
Scheme 1		Full section anchor bolt with a Φ24 × *L*2400 mm and a spacing of 800 × 800 mm between rows; Three top anchor cables with a Φ22 × *L*4300 mm and a spacing of 800 × 800 mm between them
Scheme 2		Full section anchor bolt with a Φ24 × *L*2400 mm and a spacing of 800 × 800 mm between rows; Full section anchor cable with a diameter of Φ 22 × *L*5300 mm and a spacing of 800 × 800 mm between rows
Scheme 3		Full section anchor bolt with a Φ24 × *L*2400 mm and a spacing of 800 × 800 mm between rows; Full section anchor cable with a diameter of Φ 22 × *L*5300 mm and a spacing of 800 × 800 mm between rows. 2 arch anchor cables, 3 support anchor cables with a Φ 22 × *L*7300 mm, and a spacing of 800 × 800 mm between rows

**Fig. 6:**
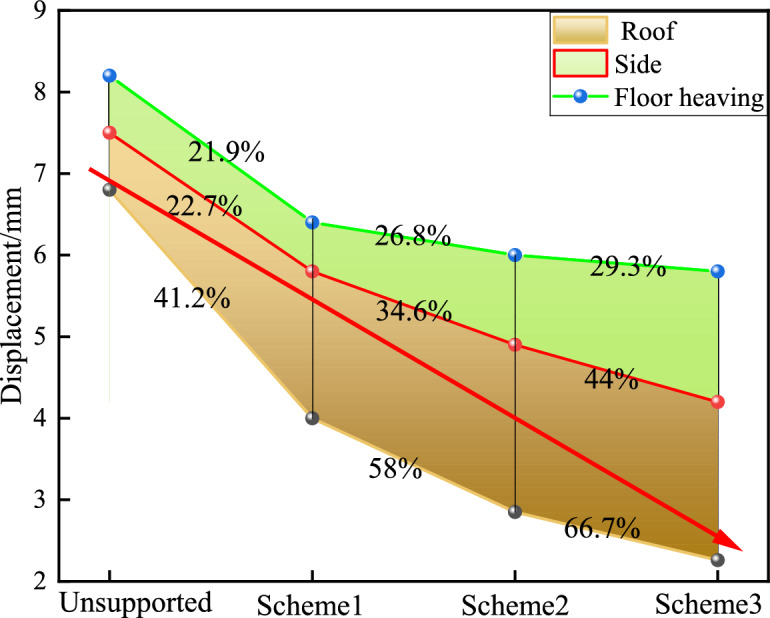
Support effects under different top support conditions.

Under the condition of unsupported floor heave of different strengths on the roof of the roadway, the range of plastic zone damage on the roof of the roadway is reduced in Fig. 6. In the unsupported state, the plastic zone depth is less than 6.8 m. The maximum depths of the plastic zone on the roof of schemes 1 to 3 are 4.0, 2.85, and 2.26 m, respectively. The maximum depths of the plastic zone in the upper part are (unsupported 7.5 m) 5.8, 4.9, and 4.2 m, respectively. The maximum depths of the plastic zone on the floor heave (unsupported 8.2 m) are 6.4, 6.0, and 5.8 m, which decrease by 21.9%, 26.8%, and 29.3%, respectively. Under the condition of unsupported floor heave, as the strength of the top support increases, the range of the plastic zone in the top support decreases significantly, whereas the decrease in the plastic zone in the floor heave is relatively small^28,29^. This indicates that increasing the strength of the top support has a smaller inhibitory effect on the plastic zone in the floor heave.

### Roadway floor support

**Fig. 7:**
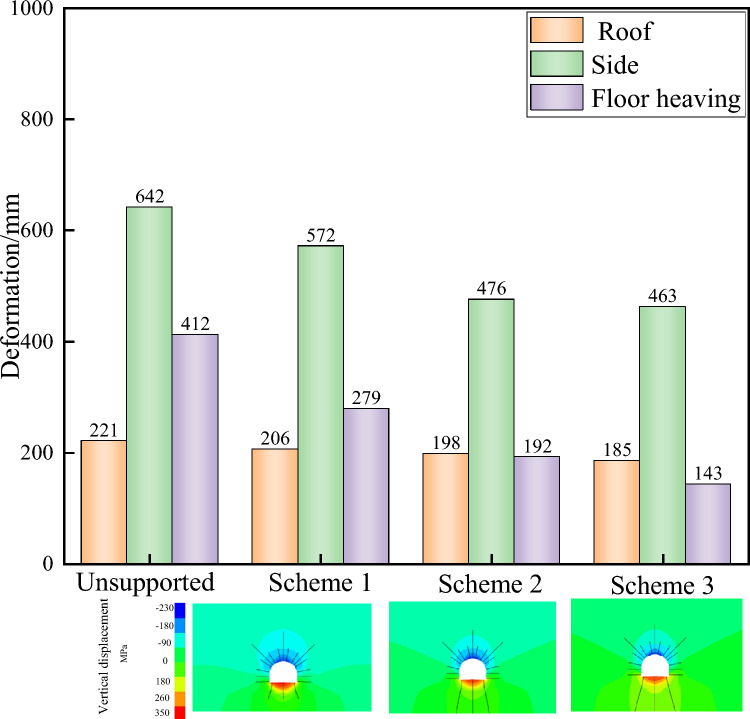
Displacement under different support schemes for floor heave.

A comparison of the support effects of different floor heave support conditions, as shown in Fig. 7 and Table 3, reveals that with increasing floor heave support strength, the amount of bottom bulging significantly decreases. The corresponding floor heave amounts for schemes 1 to 3 are 279, 192, and 143 mm, which are 32.3%, 53.4%, and 65.3% lower than those without support (412 mm), respectively. The change in roof subsidence is relatively small, whereas the displacement of the two sides changes significantly. The two sides corresponding to schemes 1 to 3 have displacements of 572, 476, and 463 mm, respectively, which are 10.9%, 25.9%, and 27.9% lower than those before support (642 mm). Therefore, improving the floor heave support has a relatively small impact on controlling the roof subsidence and promotes the deformation of the floor heave and roadway sides^30,31^.

**Table 3:** Different support schemes for the bottom.

Scheme	Simulation diagram	Describe
Scheme 1		Bottom anchor rod with *Φ* 24 × *L*3000 mm and a spacing of 1500 × 1000 mm between rows
Scheme 2		Bottom anchor cable Φ 22 × *L*730 mm, spacing between rows 1500 × 1000 mm
Scheme 3		Bottom anchor 3 Φ 22 × *L*10300 mm, spacing between rows 1500 × 1000 mm

**Fig. 8:**
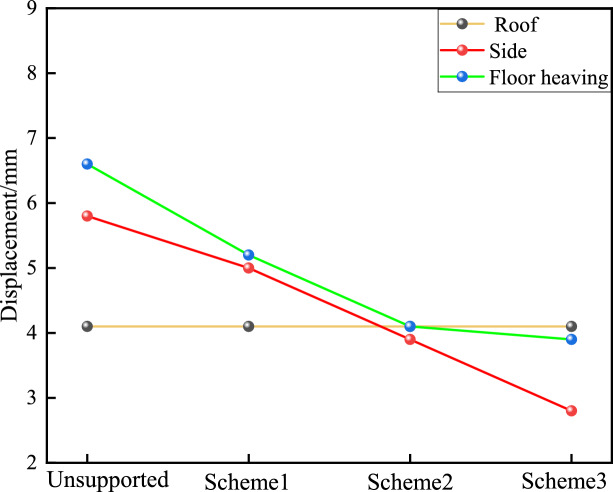
Support effects under different bottom support conditions.

The maximum depths of the plastic zone in the floor heave of schemes 1 to 3 are (6.6 m without support) 5.2, 4.1, and 3.9 m, respectively, with corresponding reductions of 21.5%, 37.9%, and 40.9%, respectively in Fig. 8. The maximum depths of the plastic zone in the upper part are (5.8 m without support) 5.0, 3.9, and 2.8 m, respectively. The maximum depth of the plastic zone in the top plate is (4.1 m without support) 4.1 m. Therefore, increasing the strength of the floor heave support can reduce the depth of damage in the plastic zone of the floor heave and both sides but has little effect on the depth of damage in the plastic zone of the top plate^32^. As the strength of the floor heave support increases, the concentrated stress in the floor heave is transferred to the top plate, reducing the stress on the floor heave and the upper part and suppressing the floor heave^33^. Meanwhile, the plastic zone of the assistance department expands. Therefore, collaborative coupling control should be adopted for the top and bottom supports, and the strength of the bottom support should match that of the top support, which can better control the deformation of the floor heave and maintain roadway stability.

Improving the strength of floor heave support has a positive effect on controlling the stability of floor heave and roadway support and has a relatively small effect on suppressing the development of plastic zones and the degree of roof deformation. Under conventional top support conditions, strong bottom support has a better control effect than ordinary support does, improving the strength of the bottom support. On the one hand, strong bottom support can reduce the degree of deformation and plastic zone damage depth of the floor heave and two sides. On the other hand, strong bottom support can promote the concentrated stress of the floor heave to move towards the top side, improve the stress of the floor heave, and reduce the degree of floor heave compression. Therefore, collaborative control should be adopted for the top, bottom, and sides to improve the strength of the roof and two sides while also increasing the support strength of the floor heave so that the rock surrounding the roadway forms a mutually influential and interactive bearing body.

### Effect of the pressure relief groove

The size of the pressure relief groove directly affects the pressure relief of the roadway floor^34^. If the design size is too small, the deformation space will close too early, resulting in insufficient pressure relief and failure to achieve the expected effect. If the design size is too large, the degree of rock fragmentation increases and the roadway may deform or even break under lateral stress in Table 4.

**Table 4:** Scheme for slotting roadway floor heave.

Scheme	Width/mm	Height/mm
Scheme 1	400	600
Scheme 2	600	800
Scheme 3	800	1000
Scheme 4	10,000	1200

The deformation trend of the floor heave is basically the same under different schemes. After the coal mining face advances to the stop mining line, the deformation of the floor heave shows a trend of"rapid growth slow growth tends to stabilise". However, the final deformation of the floor heave varies greatly. The maximum deformation of the floor heave from unloading Scheme 1 to unloading Scheme 4 is 428, 409, 326, and 248 mm, respectively. Compared with the unloading scheme (floor heave amount of 509 mm), the deformation of the floor heave in Schemes 1 to 4 decreases by 15.9%, 19.6%, 35.9%, and 51.3%, respectively. The deformation difference between Scheme 3 and Scheme 4 is relatively small. On the basis of Plan 3, increasing the size of the pressure relief groove has a smaller impact on the deformation of the floor heave but instead increases the excavation work of the pressure relief groove. Therefore, Plan 3 is more economical and reasonable.

**Fig. 9:**
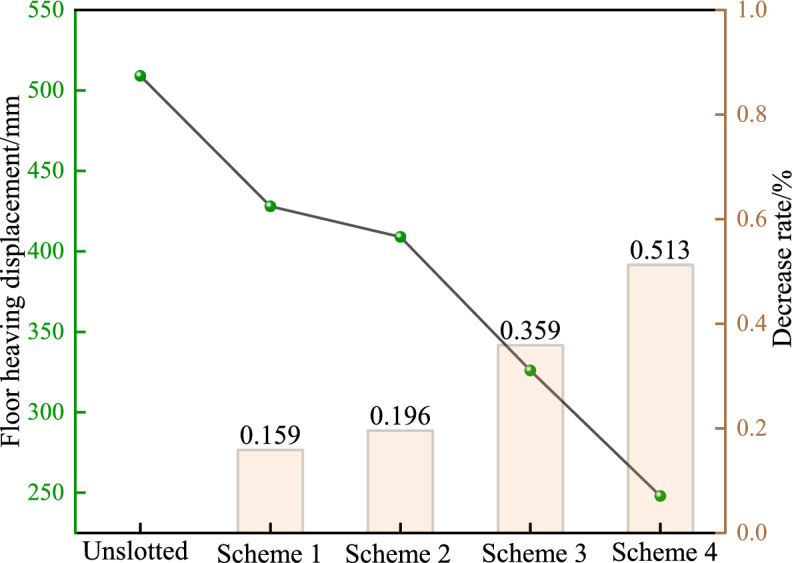
Deformation curves of roadway floors under different pressure relief conditions.

**Fig. 10:**
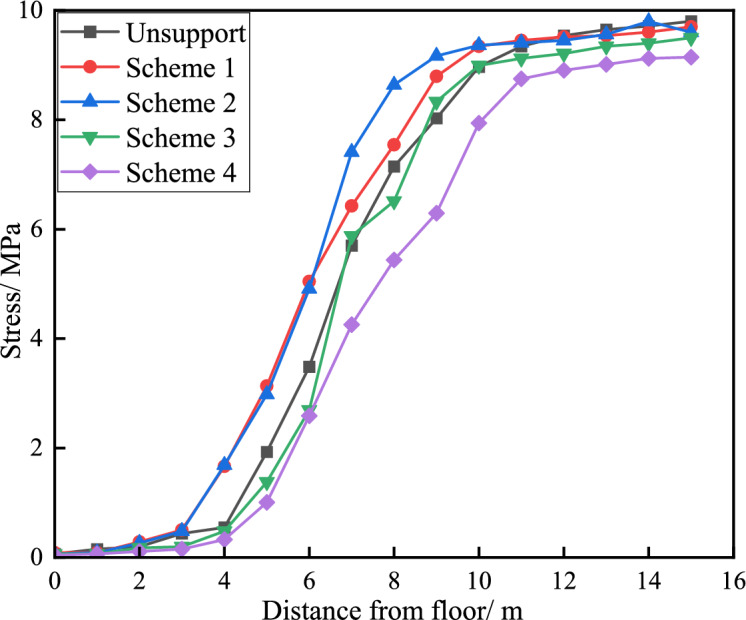
Distribution of vertical stress under different unloading pressures.

**Fig. 11:**
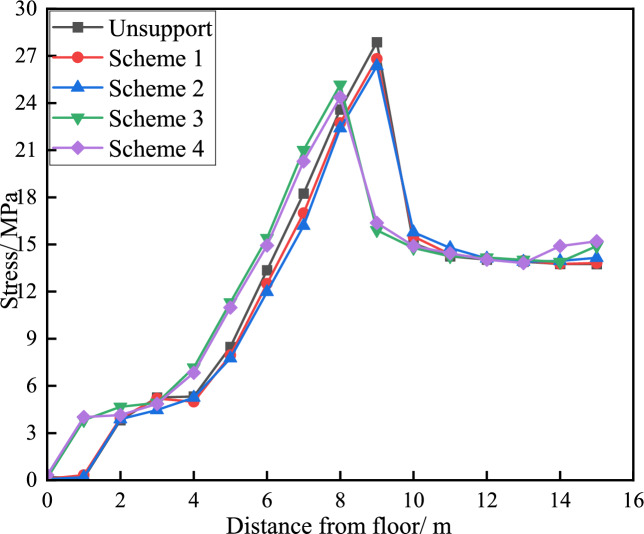
Distribution of horizontal stress under different unloading pressures.

By arranging stress measurement points in the middle of the roadway floor, after the excavation of the pressure relief groove, as the depth of the floor increases, the vertical stress shows a trend of"first increasing and then stabilising", while the horizontal stress shows a trend of"first increasing to the peak and then decreasing to the original rock stress"in Fig. 9, Fig. 10, Fig. 11. After the excavation of the pressure relief groove, the vertical and horizontal stresses of the floor heave within a depth of 9 m decrease significantly. From pressure relief Plans 1 to 4, the vertical stresses of the floor heave at a depth of 9 m were 7.5, 7.4, 6.8, and 6.5 MPa, respectively. The peak horizontal stress values of the floor heave at a depth of 9 m were 25.1, 24.7, 23.4, and 22.8 MPa, respectively, which were higher than those before pressure relief (peak value of 26.1 MPa). According to the pressure relief values, as the size of the pressure relief groove increased, the degree of stress reduction gradually increased, with Schemes 3 and 4 showing more significant reductions.

### Coupling control mechanism for unloading and fixing the roadway floor

On the basis of the numerical simulation analysis results of pressure relief and reinforcement of the roadway floor, unloading reinforcement coupling support technology with"moderate pressure relief“and”strengthened support"as the core is summarised and proposed, which forms a deep dynamic pressure roadway floor collaborative coupling control technology system that adapts to the stress and deformation of the floor through the coordinated coupling control of the top, bottom, and floor; moderate pressure relief of the floor; and multilevel coupling reinforcement control of the floor, effectively controlling the deformation and failure of the floor to keep the floor in a stable state^35–38^.

(1) Mechanism of top and side collaborative coupling support: Due to the different mechanical properties of the surrounding rock in the fractured, plastic, and elastic zones, by effectively controlling the development of the fractured and plastic zones of the top plate and two sides, the pressure of the top plate and two sides on the floor heave and the actual span of the floor heave can be reduced, which helps to alleviate floor heave. Strengthening the control of floor heave can help alleviate the deformation and damage to the two sides and top. Therefore, different lengths of anchor cables (ordinary, medium length, and long anchor cables) are used to control the shallow and deep parts of the roof surrounding rock, forming three layered reinforcement rings with gradually decreasing support strength, coupling support strength with the mechanical properties of the surrounding rock, suppressing the development of fractured and plastic zones, and providing conditions for floor heave support.

(2) Bottom pressure relief mechanism: When the coal mining face is far from the stopping line and the roadway is not affected by dynamic pressure, a pressure relief groove is excavated at the bottom corner of the roadway to weaken the control of the surrounding rock at the bottom corner. When the working face approaches the stopping line, stress is concentrated at the bottom corner of the roadway due to the influence of advanced support pressure and horizontal structural stress, which promotes the failure of the surrounding rock at the bottom corner and transfers the high stress in the shallow part of the floor heave to the deep rock outside the pressure relief groove, thus producing two protective effects: one is that the deep rock mass is in a three-dimensional stress state, has high strength, can withstand high concentrated stress, and is in a self-stable state; the second is that under the support and protection of deep rock masses, the difficulty of controlling the low stress state of shallow rock masses is reduced, and the pressure relief groove can also provide deformation release space for shallow surrounding rock, thereby reducing the deformation of the floor heave.

## Multiple-seam repeated mining centralised roadway bottom floor control technology

### Test scheme

The pressure relief grooves in tunnels with floor problems are excavated, allowing the stress around the roadway to move towards deeper rock layers, controlling the stability of the rock surrounding the roadway and achieving a small effect of pressure relief. Simultaneously, space also exists for displacement of the roadway, thereby controlling the displacement of the roadway. On the basis of the actual situation on site and the characteristics of roadways, a technical solution for the joint control of excavation pressure relief grooves, gangue filling, bottom corner anchor rods, and grouting is proposed. The specific construction sequence is as follows: undercover floor heave → excavation of pressure relief groove → filling of gangue → pouring of drainage ditch → bottom corner anchor → grouting.

**Fig. 12:**
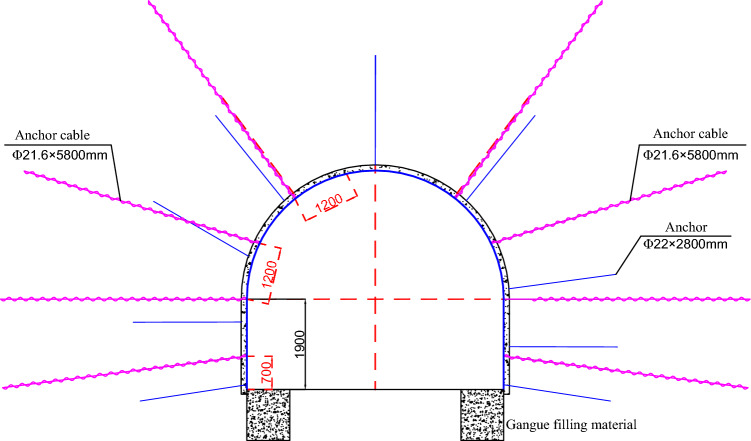
Schematic diagram of the support scheme.

The specific construction is as follows in Fig. 12:

1) Undercover the floor heave and excavate it to the original horizontal height before excavating the pressure relief groove.

2) The excavation of the pressure relief groove is carried out via a lane repair machine. A 0.8 × 1 m deep groove is excavated on the north side according to the design dimensions, and the remaining broken surrounding rock is transported out. Drain any water that has accumulated in the groove.

3) Fill the groove with crushed stones, ensuring a particle size of 25–40 mm, and compact them simply.

4) Two anchor rods are installed at the bottom corner of the tunnel, with a spacing of 800 mm and an angle of 15° between the anchor rods and the tunnel ground. Inject grout into the rock surrounding the roadway as a whole, with a spacing of 1600 × 1600 mm between the grouting pipes at the roadway support and roof and an angle of 15° between the grouting pipes at the bottom corner of the roadway support and the horizontal plane.

### Analysis of the results

After the construction of the pressure relief groove and the completion of construction, surface displacement monitoring was carried out on the roadway for 60 days. The monitoring data include the top and floor heaves of the roadway, displacement of the two sides, and amount of bottom bulging.

**Fig. 13:**
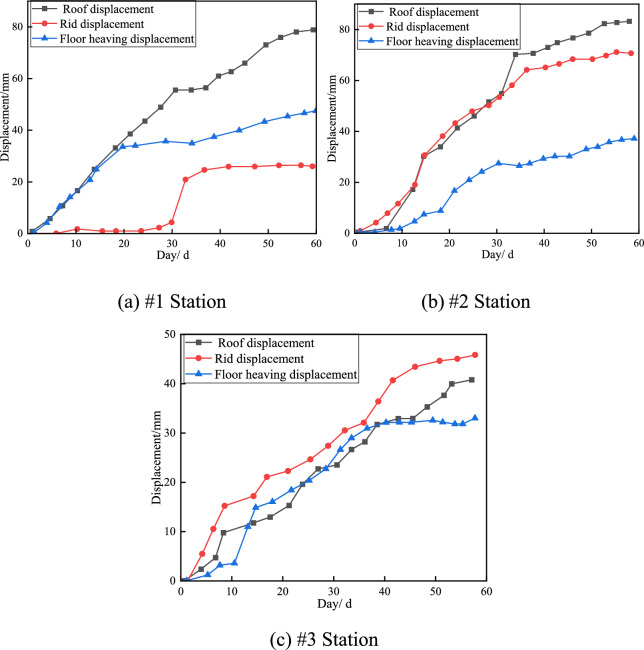
Roadway displacement monitoring.

The monitoring data during and after the construction of the pressure relief groove excavation test section of the roadway are shown in Fig. 13. During the excavation process of the pressure relief groove, the deformation of the rock surrounding the roadway shows a stable increasing trend. When the deformation of the roadway is monitored for approximately 30 d, the stress of the rock surrounding the roadway is redistributed, and significant deformation of the roadway roof, two sides, and floor occurs; that is, the roadway roof experiences a sharp subsidence phenomenon, whereas the deformation of the roadway floor shows a small increase. When the deformation of the roadway is monitored for approximately 50 days, the deformation of the surrounding rock tends to stabilise. In the process of monitoring the deformation of the surrounding rock in this tunnel, the relative displacement of the two sides of the roadway was 75 mm, the relative displacement of the roof and floor was 43 mm, and the bottom bulge was 30 mm. The deformation of the surrounding rock in the tunnel was within a reasonable range.

During the excavation process of the pressure relief groove, owing to the first excavation near measurement stations #1 and #2, the bottom plate of the roadway near measurement station #3 was in a pressure relief state, the top plate of measurement station #3 showed a significant downwards trend, and the corresponding roadway bottom plate also showed an irregular movement trend due to the pressure relief effect of the nearby pressure relief groove. When the deformation of the roadway is monitored for approximately 30 days, the stress in the rock surrounding the roadway is redistributed, and the deformation of the roadway floor is more obvious. The deformation of the roadway floor subsequently tends to stabilise. When the deformation of the roadway is monitored for approximately 50 days, the deformation of the surrounding rock tends to stabilise.

## Conclusions

1. The actual mining situation, support methods, and floor lithology of coal mine roadways with floor heaves were analysed. The roadways are located in multiple coal seams with repeated mining, and the degree of stress concentration is relatively high. The support strength of the two sides of the roadway and the roof is relatively high, and the floor is located in the weak support structure area. The corresponding roadway floor is composed of low-strength rock, which is prone to deformation and failure under high stress.
2. In response to actual mining-induced damage to the bottom of the roadway, combined with the evolution mechanism of the overlying roof structure of multiple coal seams, the factors influencing the bottom of the roadway caused by repeated mining of multiple coal seams were established. As the number of coal seam mining layers and mining depth increase, the stress concentration around the roadway gradually increases. The mechanism of deformation and failure of the roadway floor under high vertical stress caused by repeated mining of multiple coal seams was analysed. The increase in vertical stress on the two sides of the roadway corresponds to an increase in the depth of damage to the roadway floor.
3. During the repeated mining process of close-range coal seam groups, the surrounding rock experiences dynamic changes, which are reflected in the repeated breaking of the overlying rock, causing the size and position of the fragmented rock blocks to gradually shrink and become disordered, greatly reducing the possibility of forming a structure. On the basis of existing floor heave control technology, different roadway floor heave control schemes are formulated, and FLAC simulation software is used to simulate and study the stress changes and displacement deformation laws of the rock surrounding the roadway in each scheme. The optimisation scheme for controlling roadway floor heave is determined by slotting the bottom corners on both sides of the roadway, corresponding to a width of 0.8 m and a depth of 1.0 m, which is based on the actual situation on site.
4. The bulging amount of the roadway bottom in the pressure relief groove test section was 43 mm, whereas the bulging amount of the roadway bottom in the concrete inverted arch test section was only 30 mm. Considering the comprehensive economic perspective and construction difficulty, the support scheme of bottom plate slotting and pressure relief is more suitable for the bottom drum control work of the + 400 m centralised track roadway.

## Data Availability

The data that support the findings of this study are available from the author H.Y.L.
